# 17*α*-Ethynyl-5*α*-androstane-3*α*, 17*β*-diol Treatment of MNU-Induced Mammary Cancer in Rats

**DOI:** 10.4061/2011/618757

**Published:** 2011-02-14

**Authors:** Clarence N. Ahlem, James M. Frincke, Steven K. White, Christopher L. Reading, Richard J. Trauger, Rajkumar Lakshmanaswamy

**Affiliations:** ^1^Harbor BioSciences, Inc., 9171 Towne Centre Drive, Suite 180, San Diego, CA 92122, USA; ^2^Center of Excellence in Cancer Research, Department of Biomedical Sciences, Texas Tech University Health Sciences Center, 4800 Alberta Ave, El Paso, TX 79905, USA

## Abstract

N-methyl-N-nitrosourea (MNU) induces estrogen-dependent mammary tumors in female Lewis rats. We explored the antineoplastic activity of a synthetic androstane derivative, 17*α*-ethynyl-5*α*-androstane-3*α*, 17*β*-diol (HE3235), as a single agent or in combination with docetaxel compared to tamoxifen, anastrazole, and docetaxel monotherapies against MNU-induced mammary tumors in female Lewis rats. Treatment with HE3235 alone rapidly reduced tumor burden, similar in effect to tamoxifen and anastrozole. The combination of HE3235 with docetaxel was more effective than any single agent, although without apparent toxicity. Only HE3235 or HE3235 plus docetaxel continued to suppress tumor growth after cessation of treatment. HE3235 treatment increased immunohistochemical markers of apoptosis and expression of proapoptotic genes and estrogen receptor beta and decreased expression of antiapoptotic genes, androgen receptor, and estrogen receptor alpha. These data warrant clinical investigation of HE3235 for breast cancer treatment.

## 1. Introduction

Breast cancer is one of the most common cancers among women, and the incidence of breast cancer is increasing worldwide [1]. Approximately 200,000 women are diagnosed with breast cancer annually, with an associated mortality of 40,000 in the United States [2]. Currently, there are few treatments for hormone-dependent breast cancer, with tamoxifen and anastrazole being the most widely used therapies [3]. Although generally well tolerated, these treatments can be associated with significant morbidity [4], and development of resistance is common [5, 6].

The carcinogen N-methyl-N-nitrosourea (MNU) induc-es hormone-dependent mammary tumors in rats. This model has previously been used to develop tamoxifen therapy in women with breast cancer [7], and is considered to be appropriate for studies of novel compounds potentially useful for the treatment of breast cancer. Substantial evidence suggests that this rodent model system mimics human breast cancer: the initiation of cancer occurs primarily at the same site in both humans and rats, the majority of the tumors express estrogen and progesterone receptors, and tumor development is dependent on the reproductive history, diet, and hormonal milieu [8]. Thus the model provides an opportunity to examine cause-and-effect relationships of the *in situ* environment fully impacted by systemic factors [9]. 

17*α*-ethynyl-5*α*-androstane-3*α*, 17*β*-diol (HE3235) is a synthetic androstane derivative that inhibits 5-androstene-diol [10], testosterone, and estrogen (unpublished) stimulated prostate cancer cell line (LNCaP) proliferation and was thus selected as preclinical candidate for evaluation against hormone sensitive breast cancer. A well-defined molecular basis for the apparent mechanism(s) of action of HE3235 has not been elucidated. In human prostate cancer xenografts, HE3235 does not transactivate the androgen receptor (AR) and does not antagonize the action of testosterone on AR, but stimulates the androgen response element and PSA expression while decreasing AR expression, inducing apoptosis, and inhibiting androgen synthesis [10, 11]. The pharmacological characteristics of HE3235, including the nuclear hormone interaction profile of HE3235 and its major metabolites have been published [12]. HE3235 and metabolites 17*α*-ethynyl-5*α*-androstane-3*β*, 17*β*-diol, and 17*α*-ethynyl-17*β*-hydroxy-5*α*-androstan-3-one, are not potent sex hormones compared to estradiol and testosterone but have the potential to bind and transactivate AR, estrogen receptor alpha (ER*α*), and estrogen receptor beta (ER*β*) to various degrees, providing the potential for complex interactions in cells that possess nuclear and/or membrane receptors for sex steroids. In rodent and canine toxicology studies, HE3235 was generally well tolerated, with anorexia emerging at high dose in both species [12].

After conducting pilot experiments in the MNU rat mammary tumor model that indicated antitumor activity, we evaluated HE3235 as a single agent and in combination with docetaxel on tumor growth and response durability after treatment cessation. We report here that HE3235 in the rat MNU mammary cancer model exhibits a potent and durable antitumor activity, which in our hands was superior to anastrozole and docetaxel and was not accompanied by apparent toxicity. The combination of HE3235 and docetaxel did not synergize toxicity, and the antitumor activity was found to be superior to tamoxifen monotherapy. These data provide a rationale to continue investigation of this novel drug for the potential treatment of hormone-sensitive breast cancer. 

## 2. Materials and Methods

### 2.1. Carcinogenesis

All animal procedures were conducted at Texas Tech University Health Sciences Center. The procedures were approved by the Health Sciences Center's Animal Care and Use Committee and were performed in accordance with federal and local regulations. Virgin Lewis rats were purchased from Harlan Sprague Dawley (Indianapolis, IN and San Diego, CA). The rats were housed in a temperature-controlled room with a 12-hour light and dark schedule and fed a standard lab diet with access to food and water* ad libitum*. At seven weeks of age, all rats were treated with a single intraperitoneal (IP) injection of 50 mg/kg N-methyl-N-nitrosourea (Sigma, St. Louis, MO) as previously described [13, 14]. 

### 2.2. Formulation of Test Compounds and Drug Treatment Regimens

When the rats developed palpable tumors (approximately 5 mm × 5 mm, 90 days after MNU) they were divided into seven treatment groups of thirteen animals each: (1) Control, (2) 6.6 mg HE3235 (high-dose HE3235), (3) 4 mg HE3235 (low-dose HE3235), (4) high-dose HE3235 + 1.5 mg docetaxel, (5) 1.5 mg docetaxel, (6) 2.5 mg anastrazole, and (7) 0.25 mg tamoxifen. Additional groups of animals were treated for two weeks with either vehicle or 6.6 mg HE3235 (*N* = 10/group) for purposes of the histopathology evaluation, immunohistochemical staining, and gene expression.

The doses of HE3235 were selected after conducting 28-day treatment experiments in female rats with established MNU tumors with 4 and 8 mg HE3235 per day. A complete dose titration to identify an optimal dose was not conducted. The intended high dose for the current study was 8 mg per day, but analysis of the test article indicated only 6.6 mg was achieved. Docetaxel was selected as a model taxane for combination therapy with HE3235 because of the common use of taxanes in breast cancer management and the potential for additive or synergistic activity from agents with different mechanisms of action.

Aqueous solutions of HE3235 were prepared with *β*-cyclodextrin sulfobutyl ether (Captisol, CyDex, Lenexa, Kansas). 17*α*-Ethynyl-5*α*-androstane-3*α*, 17*β*-diol (HE3235) (Hollis-Eden Pharmaceuticals, San Diego, CA) was formulated as a solution of either 20 or 33 mg/mL in 30% cyclodextrin (w/v). HE3235 was administered daily by intraperitoneal injection (IP) for 4 weeks (4 or 6.6 mg, 1 mL/kg). HE3235 treatment was combined with docetaxel by contemporaneous injection of separate test articles. Docetaxel (Taxotere, National Drug Code 0075-8001-20, Sanofi-Aventis U.S. LLC) was serially diluted with 13% aqueous ethanol and 0.9% saline according to the manufacturer's instructions to yield a 0.74 mg/mL solution, which was used immediately after preparation. Two milliliters of diluted docetaxel in saline (1.5 mg, 6 mg/kg, 8.1 mL/kg) were administered by IP injection once weekly for four weeks. Anastrazole (AK Scientific, Inc, Mountain View, CA) was prepared as a 10 mg/mL solution in 30% aqueous cyclodextrin and administered daily by IP injection (2.5 mg/day; 1 mL/kg) for four weeks. Tamoxifen (free base, Tocris BioScience, Ellisville, Mo) was prepared as a 1.25 mg/mL solution in olive oil, and administered by subcutaneous injection (SC) once weekly (0.25 mg, 1 mg/kg, 0.8 mL/kg) for four weeks. Vehicle-treated animals received 1 mL/kg 30% aqueous cyclodextrin daily for 4 weeks. The aqueous ethanol and olive oil vehicles used for docetaxel and tamoxifen, respectively, were administered only once per week and were assumed to have minimal potential to affect tumor growth.

### 2.3. Tumor Volume Measurements

Animals were palpated twice weekly beginning one month after carcinogen exposure until the end of the experiment to monitor mammary cancer development. Tumor dimensions of length (*r*_1_) and width (*r*_2_) were measured with a vernier caliper, and tumor volumes were estimated with the formula *V* = (4*π*/3)∗*r*_1_^2^∗*r*_2_ (mm^3^) [15]. The histopathology of paraffin sections was examined from one subset of animals (treated for 2 weeks) to confirm the carcinomatous nature of the palpable cancers. 

### 2.4. Immunohistochemistry

After two weeks of treatment (vehicle or 6.6 mg HE3235) and after confirmation of the carcinomatous nature of the samples, the tumor sections were stained for poly(ADP-ribose) polymerase (PARP) and estrogen receptor alpha (ER*α*) expression using standard immunohistochemistry techniques. Cell nuclei were counterstained with Mayer's hematoxylin (Sigma) and examined by light microscopy. The percentage of positively stained cells was determined by dividing the number of positively stained cells by the total number of cells counted and multiplied by 100.

### 2.5. Real-Time PCR

A small set of genes relevant to cell proliferation, apoptosis, and metastatic potential was quantified by RT-PCR: amphiregulin (Areg), androgen receptor (AR), tumor protein 53 (p53), Bcl2 antagonist of cell death (Bad), apoptosis regulator BAX (bax), B-cell CLL/lymphoma 2 (Bcl-2), caspase 3 (Casp3), caspase 8 (Casp8), caspase 9 (Casp9), Cyclin D1 (Ccnd1), estrogen receptor alpha (ER*α*), estrogen receptor beta (ER*β*), progesterone receptor isoform A (PR-A), vascular endothelial growth factor (VEGF), and *β*-actin. Total RNA was extracted from the frozen tissues using a guanidinium thiocyanate-phenol-chloroform extraction procedure and treated with DNAse [16]. RTPCR was performed on triplicate samples, using the QuantiTect Reverse Transcription Kit (Qiagen, Valencia, CA) according to the manufacturer's recommendations, and the relative quantitation of gene expression was calculated using the comparative Ct method [17]. Data are expressed as the mean fold differences compared to vehicle controls normalized to *β*-actin expression.

### 2.6. Statistical Methods

The significance of differences between means or paired values were calculated using Student's *t*-test. Tumor volumes censored for death used the last observation carried forward (LOCF) for the purpose of data analysis. The significance of treatment effects on animal survival was determined by Fisher's exact test using SAS software (Cary, NC).

## 3. Results

**Figure 1:**
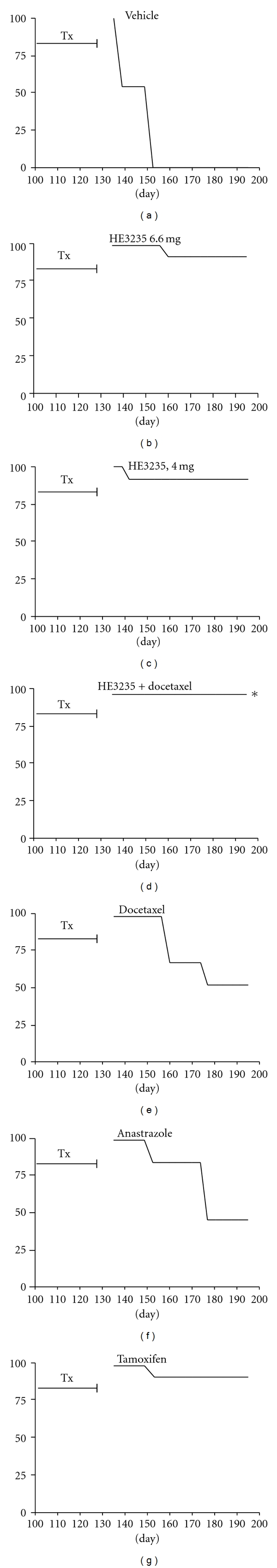
% Survival. Seven-week-old female Lewis rats were treated with a single IP injection of 50 mg/kg MNU. Tumors developed for 90 days, prior to treatment (*n* = 13) for 28 days with (a) cyclodextrin vehicle daily, (b) 6.6 mg HE3235 daily, (c) 4 mg HE3235 daily, (d) 6.6 mg HE3235 daily + 1.5 mg docetaxel weekly, (e) 1.5 mg docetaxel weekly, (f) 2.5 mg anastrazole daily, and (g) 0.25 mg tamoxifen weekly. HE3235 in combination with docetaxel was more effective than comparator monotherapies at promoting survival. *Y*-axis, percent survival; *X*-axis, study day. Therapy (Tx) started on Day 101 and ended on Day 128. The first animal was sacrificed on day 139 (vehicle group). *P = .0149 versus docetaxel on day 195.

The high dose of HE3235 and HE3235 in combination with docetaxel significantly inhibited tumor growth without apparent signs of toxicity. HE3235 in combination with docetaxel was superior to all other treatments. No animals treated with the combination of HE3235 plus docetaxel were sacrificed because of tumor burden (*P* = .0149 versus docetaxel alone); one animal was sacrificed in each of the groups treated with high or low-dose HE3235 monotherapy or tamoxifen (*P* = .073 versus docetaxel alone); six were sacrificed in the docetaxel group and seven in the anastrozole group; all animals were sacrificed in the vehicle group (Figure 1).

**Figure 2:**
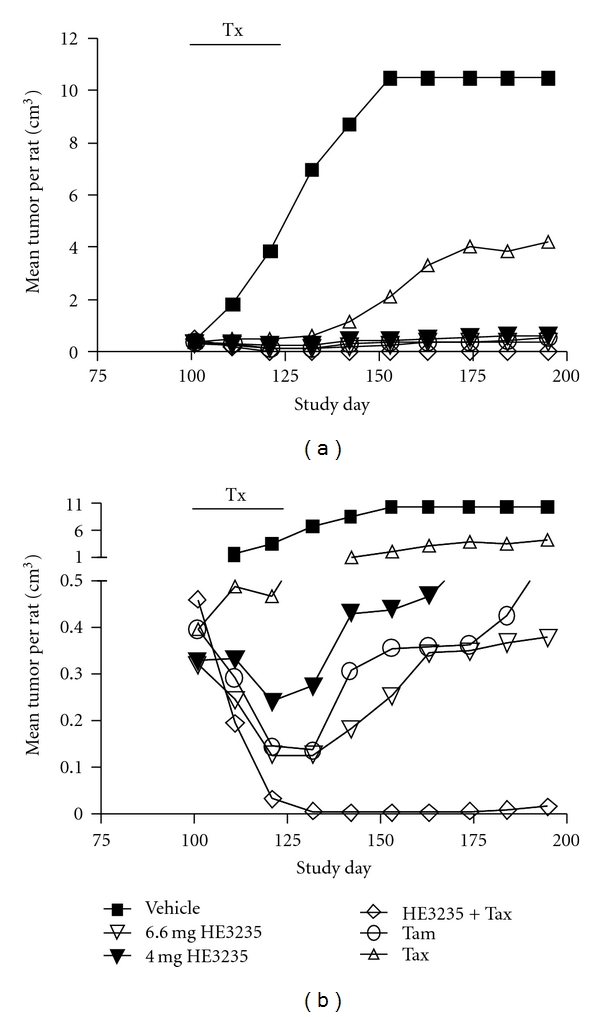
Tumor volume in response to treatment. Seven-week-old female Lewis rats were treated with a single IP injection of 50 mg/kg MNU. Tumors developed for 90 days, prior to treatment (*n* = 13) for 28 days with (1) cyclodextrin vehicle daily, (2) 6.6 mg HE3235 daily, (3) 4 mg HE3235 daily, (4) 6.6 mg HE3235 daily + 1.5 mg docetaxel weekly, (5) 1.5 mg docetaxel weekly, (6) 2.5 mg anastrazole daily, and (7) 0.25 mg tamoxifen weekly. HE3235 in combination with docetaxel was more effective than comparator monotherapies at decreasing the mean tumor volume per animal. Upper graph, all results plotted full scale; lower graph, split and expanded *Y*-axis. Anastrazole results were similar to docetaxel, but not plotted to improve clarity. The mean tumor volume for vehicle on day 101 was 0.41 cm3, which was not plotted to improve clarity. Tax, docetaxel, Tam, tamoxifen.

All animals had at least one palpable tumor at initiation of therapy. On the first day of treatment (Day 101), the mean tumor volume was 0.38 ± 0.05 mm^3^ (range 0.31 mm^3^ (anastrazole group) to 0.46 mm^3^ (high-dose HE3235-docetaxel combination)). Tumors in vehicle-treated animals grew rapidly, with animals sacrificed for humane reasons in this group beginning on Day 139, and the last animal euthanized on Day 153. Consistent with a pilot experiment, treatment with high-dose HE3235 alone had a rapid and potent antitumor effect as indicated by a steep decline in the tumor volume after initiation of treatment (Figure 2). The cytoreductive activity of all active treatment groups was similar for the first two weeks of therapy, but the tumor ablative activity of low-dose HE3235, docetaxel, and anastrazole waned during the second half of the treatment period. In addition, tumor volume increased substantially in the docetaxel and anastrazole groups during the observation period after treatment cessation. None of these three treatments showed statistically significant antitumor activity at the end of the treatment period compared to treatment initiation (*P* > .1). In all three instances, treatment appeared to be more effective in reducing or eliminating small tumors, while larger tumors were generally more resistant. In contrast, high-dose HE3235 (*P* = .011) or tamoxifen (*P* = .0042) aggressively ablated tumor volume through the end of the treatment period, with a modest volume increase during the observation period. High-dose HE3235 combined with docetaxel prevented tumor growth through the last day of observation (Day 195) and was more effective at the end of treatment than either agent used separately (*P* = .0113 versus high-dose HE3235 and *P* = .0390 versus docetaxel). The mean tumor burden in the combination therapy group was not significantly different from tamoxifen at the end of treatment (*P* = .3451) or at the end of the observation period (*P* = .1383).

**Figure 3:**
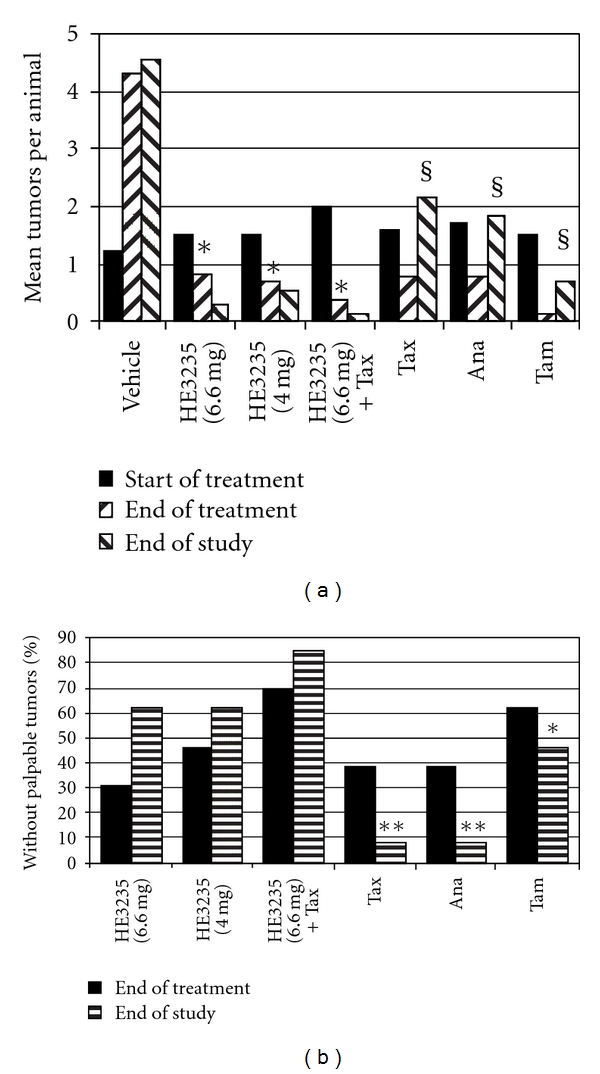
Tumor Incidence. Seven-week-old female Lewis rats were treated with a single IP injection of 50 mg/kg MNU. Tumors developed for 90 days, prior to treatment (*n* = 13) for 28 days with (1) cyclodextrin vehicle daily, (2) 6.6 mg HE3235 daily, (3) 4 mg HE3235 daily, (4) 6.6 mg HE3235 daily + 1.5 mg docetaxel weekly, (5) 1.5 mg docetaxel weekly, (6) 2.5 mg anastrazole daily, and (7) 0.25 mg tamoxifen weekly. HE3235 as a monotherapy or in combination with docetaxel, was more effective at decreasing the number of tumors and rendering animals disease-free, than comparator therapies. (a) the effect of treatment on the average number of tumors per animal. *P start versus end of treatment =  .0008 (6.6 mg HE3235),  .0007 (4 mg HE3235), and <.0001 (HE3235+Tax); ^§^ *P* end of treatment versus end of study  =  .0003 (Tax),  .0016 (Ana), <.0001 (Tam). (b) the percentage of rats in each group without palpable tumors. (there were no disease-free animals in the vehicle group, not plotted.). **P versus HE3235 + Tax, <.0001, **P versus HE3235 + Tax,  .0405. Tax, docetaxel; Ana, anastrozole; Tam, tamoxifen.

The relative effectiveness of each treatment was scored for tumor incidence at treatment end and study end compared to baseline (Figure 3). Tumor incidence increased dramatically (from 1.23 to 4.31, *P* = .0001) in the vehicle-treated-group during the dosing period (Day 101 to 128) and, as expected, decreased in response to all active treatments (*P* < .05). Tumor incidence in the monotherapy groups was not significantly higher than the HE3235-docetaxel combination at treatment end, except for docetaxel (*P* < .0089). After cessation of dosing, tumor incidence increased sharply in the docetaxel (0.92 to 2.1, *P* = .0051) and anastrazole (0.69 to 1.77, *P* = .0051) groups and increased slightly in the tamoxifen group (0.15 to 0.77, *P* = .0362), while the tumor incidence with HE3235 monotherapy or docetaxel combination continued to decline, although not statistically lower than at treatment end. 

The incidence of disease-free animals in the HE3235 and tamoxifen groups was comparable at treatment end. Eight tamoxifen rats were disease free, compared to six and seven in the low- and high-dose HE3235 groups respectively, and ten in the HE3235-docetaxel group (Figure 3). At the end of the observation period, tumor incidence increased by two in the tamoxifen group (6 of 13 animals were disease free), whereas tumor incidence decreased by two in the low-dose HE3235 group (8 of 13), one in the high-dose HE3235 group (8 of 13), and one in the HE3235-docetaxel group (11 of 13, *P* = .0405 versus tamoxifen; not significant versus high or low HE3235 monotherapy).

**Figure 4:**
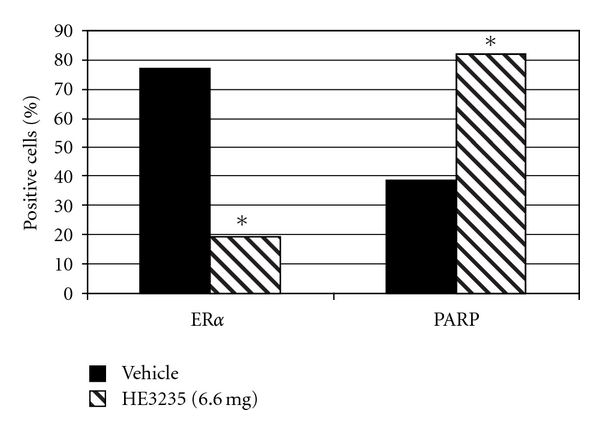
% Tumor cells positive for ER*α* and PARP. Seven-week-old female Lewis rats were treated with a single IP injection of 50 mg/kg MNU. Tumors developed for 90 days, prior to treatment (*n* = 10) for 14 days with cyclodextrin vehicle daily, or 6.6 mg HE3235 daily. Paraffin sections of tumors were examined for histopathology and stained for immunohistochemistry with antibodies to PARP or ER. HE3235 treatment increased the frequency of cells staining positive for the apoptotic maker, PARP, and decreased the frequency of ER*α*, which is associated with tumor survival. *P < .0001.

**Figure 5:**
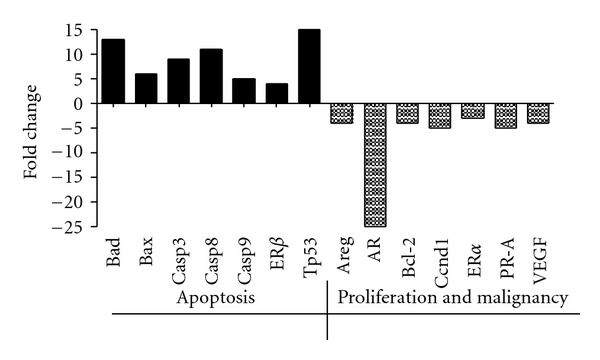
Expression of proapoptosis and differentiation genes in tumors. Seven-week-old female Lewis rats were treated with a single IP injection of 50 mg/kg MNU. Tumors developed for 90 days, prior to treatment (*n* = 10) for 14 days with cyclodextrin vehicle daily, or 6.6 mg HE3235 daily. Gene expression was measured by RT-PCR. The graphs show ratios of gene expression in HE3235 (6.6 mg) treated tumor samples relative to vehicle treated, normalized to *β*-actin. Amphiregulin (Areg), androgen receptor (AR), tumor protein 53 (p53), Bcl2 antagonist of cell death (Bad), apoptosis regulator BAX (bax), B-cell CLL/lymphoma 2 (Bcl-2), caspase 3 (Casp3), caspase 8 (Casp8), caspase 9 (Casp9), Cyclin D1 (Ccnd1), estrogen receptor alpha (ER*α*), estrogen receptor beta (ER*β*), progesterone receptor isoform A (PR-A), and vascular endothelial growth factor (VEGF). HE3235 treatment upregulates proapoptotic genes in tumors and downregulates tumor proliferation and malignancy genes.

The effects of HE3235 on tumor tissue were examined by immunohistochemistry in satellite groups treated with either vehicle or high-dose HE3235 for 2 weeks. HE3235 increased the frequency of PARP stained cells two-fold (2,590 of 3,123 (82.9%) HE3235 treated versus 1,212 of 3,079 (39.4%) vehicle treated, *P* < .0001), and decreased the frequency of ER*α* staining approximately 4-fold (603 of 3,058 (19.7%) HE3235 treated versus 2,349 of 3,051 (77.0%) vehicle treated, *P* < .0001) (Figure 4). The expression of genes associated with cell proliferation, apoptosis, and metastatic potential were consistent with the immunohistochemistry results (Figure 5). Proapoptotic genes were upregulated: Casp3 (9-fold), Casp8 (11-fold), Casp9 (5-fold), p53 (15-fold), Bad (13-fold), Bax (10-fold), and ER*β* (4-fold), while genes associated with malignancy, metastasis, and escape from treatment were downregulated: AR (25-fold), ER*α* (3-fold), PR-A (5-fold), and VEGF (4-fold). The expression of the autocrine growth factor, amphiregulin, and the anti-apoptotic protein, Bcl-2, were also decreased approximately 4-fold. 

## 4. Discussion

HE3235 is a novel androstane derivative, initially identified as an agent to treat prostate cancer. Prior studies with a hormone sensitive LNCaP cell line demonstrated that HE3235 could reduce the incidence and development of androstenediol stimulated xenografts [10]. Using the MNU-induced rat mammary cancer model, we have shown that HE3235 is also active against these estrogen-dependent tumors. This activity appears to be associated with an induction of apoptosis and suppression of androgen and estrogen (alpha) nuclear hormone receptor expression. The activity of HE3235 monotherapy was comparable to tamoxifen and superior to anastrozole. When combined with docetaxel, HE3235 was superior to both. No indication of toxicity was observed from any treatment, and the combination of HE3235 with docetaxel did not potentiate toxicity. Doses of comparitor therapies were adapted from previous reports [18–21]. A pharmacokinetics study estimated the daily exposure from the 6.6 mg dose of HE3235 to be approximately 12,765 ng∗hr/mL, which is similar to the maximum exposure without severe toxicity (including anorexia) that was observed in 28-day toxicity studies in rats and many-fold higher than observed in a Phase I/II prostate cancer clinical study [12]. HE3235 is orally bioavailable but was administered parenterally in this study (and in previous prostate cancer models) to reduce oral cyclodextrin vehicle effects. 

HE3235 aggressively shrank established tumors and prevented the appearance of new tumors. The rate of tumor volume reduction and degree of tumor suppression after treatment cessation was similar for high-dose HE3235 monotherapy and tamoxifen. When HE3235 was combined with docetaxel, not only was the tumor-ablative activity enhanced, but tumor suppression was also maintained for sixty days after treatment cessation. The potential for tumors to re-emerge in these animals was not determined, but this does not diminish the implication of a substantial increase in apparent disease-free survival.

HE3235 and its major metabolites, 17*α*-ethynyl-5*α*-androstane-3*β*, 17*β*-diol, and 17*α*-ethynyl-17*β*-hydroxy-5*α*-androstan-3-one, act as binding and/or transcriptional agonists and antagonists of AR, PR, ER*α*, and ER*β* nuclear hormone receptors *in vitro* [12]. The pharmacology of HE3235 and its metabolites in sex hormone-dependent cancers is complex. HE3235 inhibits both androgen- and estrogen-induced proliferation of LNCaP cells but does not appear to interfere with androgen response element (ARE) transcription [10]. Mechanistically, this does not exclude the possibility that HE3235 and metabolites also interact with signal transduction pathways mediated by cell surface receptors. Development of a weak ER agonist for breast cancer therapy, other than ER-negative variants such as triple negative breast cancer, must address concerns regarding the potential for proliferative effects on cancer cells. Clinical development of HE3235 must be predicated on the demonstration that proapoptotic effects dominate potential proliferative effects. Our results in the MNU model suggest that this indeed occurs, at least in rat mammary tumors, although further exploration of this issue using other tumor models may be necessary.

As reported here, HE3235 diminished AR and ER*α* expression in the rat MNU model and was previously reported to decrease AR expression in a hormone-independent prostate cancer xenograft model [11]. If HE3235 is found to be active against breast cancer in humans, decreased ER*α* and AR expression are associated with improved prognosis and reduced escape from therapy [22]. HE3235 also decreased circulating levels of sex steroids in female rats and male dogs without apparent perturbation of serum gonadotropin concentrations and decreased testosterone in LuCaP35V prostate cancer xenografts in *castrated* mice [11, 12]. However, the ability of HE3235 to elicit these activities in humans, and the relationship of these observations to HE3235's antitumor activity is not known. Docetaxel, through stabilization of microtubules [23], and HE3235, by virtue of enhancing the expression of proapoptotic genes and decreasing expression of cell survival genes, are both proapoptotic agents, and as such their combined antitumor effect was enhanced, although our studies were not designed to demonstrate synergy. The increased activity of the HE3235-docetaxel combination could also be explained by a reduction in endogenous estradiol, since estradiol can reduce taxane-induced apoptosis through activation of ERK via a plasma membrane receptor, although other ERK-independent pathways may also contribute to these estrogen mediated effects [24]. Anastrozole is a standard of treatment for reducing endogenous estradiol in breast cancer patients that failed first-line therapy. Although active in the MNU model, anastrazole was inferior to HE3235 in our study. 

Gene expression assays showed HE3235 increased expression of proapoptotic genes and decreased expression of malignancy and tumor survival genes, consistent with the immunohistochemical analysis and observed effects on tumor growth. The contrasting effect on the malignancy promoting genes, AR, PR-A, and ER*α* (downregulated), compared to the prodifferentiation maintenance gene, ER*β*, in combination with effects on apoptosis associated genes, highlights HE3235 as a differentiation agent, as it induced programmed cell death in mammary cancer (reported here) and prostate cancer models [10].

Relative to humans, rodents are known to aggressively and differentially metabolize native adrenal steroids [25, 26], but this was not observed with the synthetic androstanediol derivative HE3235. In female rats, as in male humans, the weakly estrogenic HE3235 metabolite, 17*α*-ethynyl-5*α*-androstane-3*β*, 17*β*-diol, and metabolite, 17*α*-ethynyl-17*β*-hydroxy-5*α*-androstan-3-one are present in relatively low abundance in plasma [12]. Furthermore, HE3235 is the dominant unconjugated molecular species in rats and humans, decreasing concern that the active molecular entity in the preclinical model will not be present in the clinical setting. The half-life of elimination in humans is approximately 14 hours, which is compatible with both QD and BID administration schedules; however, the half-life is only about 1-2 hours in mice or Lewis rats [12]. This disparity between the preclinical rat model and humans necessitates the use of disproportionally high doses in rodent models to compensate for the rapid elimination compared to humans.

Many different approaches are being used by clinical and experimental investigators to study prevention and treatment of breast cancer. Currently there are only three accepted treatments for breast cancer prevention in high-risk females: ovarian ablation, total mastectomy/lumpectomy, and prolonged treatment with tamoxifen. None of these are universally acceptable due to the associated physical, psychological, and physiological side effects. As far as breast cancer treatment is concerned, the options are based on the phenotypic profile of nuclear sex hormone receptors in the patient's tumors (ER*α*, PR) [27]. All treatments currently used against these targets have significant side effects, and a good share of patients elect to discontinue therapy [28, 29].

Currently, the third generation oral aromatase inhibitors are considered to be ideal candidates to either enhance the activity of tamoxifen or replace it entirely for the prevention of breast cancer recurrence in postmenopausal women. In the head-to-head arm of the ATAC (anastrozole, tamoxifen alone or in combination) trial, at the 100-month analysis, the disease-free survival advantage for the hormone receptor positive population was 4.8% in favor of anastrozole over tamoxifen [30]. Aromatase inhibitors have an overlapping, but not identical side effect profile to tamoxifen. One of the most disturbing side effects of aromatase inhibitors is the emerging effect on bone, with a significant increase in fractures during the course of the ATAC trial, and an earlier increase in bone density loss, and an even earlier increase in bone turnover markers [31]. Clearly, new drugs that treat breast cancer without these harmful side effects would have a positive impact on the management of this disease.

Significantly in the cancer therapy setting, where drugs are frequently used in combination, HE3235 does not have appreciable hepatic, hematopoietic, or cardiopulmonary toxicity at what are currently believed to be cytoreductive doses [12]. In contrast to anastrozole, HE3235 may have a positive effect on bone, as an increase in bone mineral density was found relative to vehicle in an intratibial prostate cancer xenograft study [11]. This may be relevant to breast cancer considering the high incidence of bone metastases in this disease [32]. Accordingly, the safety and activity profile of HE3235 thus provides a basis for its combination with classic cytotoxic agents, such as the taxanes. Such combinations would be expected to result in complementary antineoplastic mechanisms, with a reduced incidence of treatment escape. Breast cancer adjuvant therapy employs various combinations of anthracyclines, taxanes, and cyclophosphamide [33]. The data presented here suggest that if the activity in rodents is present in humans, the addition of HE3235 to an adjuvant treatment regime may significantly enhance the therapeutic benefit. With its favorable nonclinical safety profile and a novel mechanism of action, HE3235 is an interesting drug candidate for evaluation against hormone-sensitive breast cancer.
